# High-Throughput Transcriptomics Differentiates Toxic versus Non-Toxic Chemical Exposures Using a Rat Liver Model

**DOI:** 10.3390/ijms242417425

**Published:** 2023-12-13

**Authors:** Venkat R. Pannala, Michele R. Balik-Meisner, Deepak Mav, Dhiral P. Phadke, Elizabeth H. Scholl, Ruchir R. Shah, Scott S. Auerbach, Anders Wallqvist

**Affiliations:** 1 Department of Defense Biotechnology High Performance Computing Software Applications Institute, Telemedicine and Advanced Technology Research Center, U.S. Army Medical Research and Development Command, Fort Detrick, Frederick, MD 21702, USA; 2 The Henry M. Jackson Foundation for the Advancement of Military Medicine, Inc., Bethesda, MD 20817, USA; 3 Sciome LLC, Research Triangle Park, Durham, NC 27709, USA; michele.balik-meisner@sciome.com (M.R.B.-M.); deepak.mav@sciome.com (D.M.); dhiral.phadke@sciome.com (D.P.P.); elizabeth.scholl@sciome.com (E.H.S.); ruchir.shah@sciome.com (R.R.S.); 4 Division of Translational Toxicology, National Institute of Environmental Health Sciences, Research Triangle Park, Durham, NC 27709, USA; auerbachs@niehs.nih.gov

**Keywords:** high-throughput transcriptomics, liver toxicity, gene expression, histopathology, dose response

## Abstract

To address the challenge of limited throughput with traditional toxicity testing, a newly developed high-throughput transcriptomics (HTT) platform, together with a 5-day in vivo rat model, offers an alternative approach to estimate chemical exposures and provide reasonable estimates of toxicological endpoints. This study contains an HTT analysis of 18 environmental chemicals with known liver toxicity. They were evaluated using male Sprague Dawley rats exposed to various concentrations daily for five consecutive days via oral gavage, with data collected on the sixth day. Here, we further explored the 5-day rat model to identify potential gene signatures that can differentiate between toxic and non-toxic liver responses and provide us with a potential histopathological endpoint of chemical exposure. We identified a distinct gene expression pattern that differentiated non-hepatotoxic compounds from hepatotoxic compounds in a dose-dependent manner, and an analysis of the significantly altered common genes indicated that toxic chemicals predominantly upregulated most of the genes and several pathways in amino acid and lipid metabolism. Finally, our liver injury module analysis revealed that several liver-toxic compounds showed similarities in the key injury phenotypes of cellular inflammation and proliferation, indicating potential molecular initiating processes that may lead to a specific end-stage liver disease.

## 1. Introduction

Toxicogenomics, the application of transcriptomics, together with protein and metabolic profiling, to study how cells respond to environmental and chemical exposures by measuring changes in gene, protein, and metabolite expression profiles, has proven to be a valuable tool for environmental risk assessment [1]. These technologies offer a wealth of mechanistic insights and have transformed our knowledge of cellular and molecular pathways to establish a link between exposure and adverse outcomes [2,3]. However, low throughputs have precluded thorough evaluations of concentration responses and chronological effects, which are essential to gain mechanistic insights and predictive capabilities [4]. Liver injuries due to toxicant exposures remain one of the leading causes of failed therapeutics and continue to be a common cause of death in the United States [5,6]. Several transcriptomic studies have attempted to characterize liver injuries in response to chemical exposures at the molecular level; however, the complexities of biological responses in terms of dose responses and temporal progression have largely prevented the prediction of toxic outcomes [7,8,9,10].

High-throughput transcriptomics (HTT) can provide biomarkers that predict specific adverse outcomes and establish points of departure at which key molecular events occur following exposures. For example, whole-genome transcriptomics can help link perturbations, such as chemical exposures, with changes in biological processes that result in a specific toxic endpoint or disease phenotype. However, performing whole transcriptomics, such as RNA sequencing, may not be cost-effective when the sample size is very large or when testing a wide spectrum of compounds. To circumvent this problem, The National Toxicology Program (NTP) has pioneered the development of a novel transcriptomic gene set to infer the transcriptional profiles of genes without measuring every individual gene in a transcriptome [11,12]. The program created a sentinel gene list named S1500+, combining both gene co-expression-based information from large-scale transcriptomics studies and genes nominated by scientists with expert knowledge in toxicology and other related fields.

The NTP used 5-day rat in vivo exposure studies and leveraged the newly developed HTT platform as an alternative data stream for understanding a quantitative estimate of chemical hazard as a bioactivity-based bridge between traditional apical endpoints and HTT data generated from toxic exposures [13]. Using a set of 18 well-studied chemicals with established toxicity data from chronic studies of rats and mice, they performed 5-day in vivo chemical exposure studies with male rats and evaluated whether the transcriptional benchmark dose (BMD) value for the liver and kidneys after 5 days of oral exposure in rats could estimate the lowest histopathological BMD value that was observed in chronic or sub-chronic toxicity studies. The study showed that for many of the chemicals, the lowest transcriptional BMD estimated from HTT was within a factor of five of the values estimated from the traditional apical endpoints, and this suggested that the 5-day rat in vivo model provides reasonable estimates of BMD values for predicting traditional apical endpoints.

In this study, we further explored the 5-day rat in vivo HTT model as a predictive tool using the data from the 18 environmental chemicals studied by the NTP. Using the chronic liver toxicity study outcomes observed for these chemicals [13], we divided them into two categories: non-hepatotoxic and hepatotoxic chemicals. Furthermore, since the S1500+ dataset contains only ~2500 genes compared to the whole rat genome of 20,000 genes, we extrapolated the gene expression changes from the S1500+ dataset to the whole transcriptome to be able to include information from all genes. We then analyzed how alterations in the extrapolated gene expression profiles obtained from the HTT platform can provide useful molecular signatures or mechanistic insights for depicting the predefined toxicity outcomes identified in the chronic studies. Specifically, along with the traditional statistical methods to analyze the significant changes in genes and KEGG pathways, we used our liver injury module approach, which predicts histopathological phenotypic outcomes based on gene expression data. Our analysis of the changes in gene expression revealed a distinct expression pattern at higher dose levels for the hepatotoxic chemicals compared to the non-hepatotoxic chemicals. Furthermore, we identified a set of common genes that changed significantly across the hepatotoxic chemicals at the highest concentration, and their expression pattern suggested that most of these common genes were upregulated for the hepatotoxic chemicals compared to the non-hepatotoxic chemicals, indicating potential gene signatures that can be monitored to predict hepatotoxicity. Furthermore, our liver injury module analysis predicted potential histopathological outcomes for each of the chemicals studied and indicated that hepatotoxic chemicals showed molecular processes in cellular inflammation and proliferation as the major histopathological changes that lead to liver toxicity.

## 2. Results and Discussion

### 2.1. Gene Expression Patterns in Dose–Response Studies Predict Toxicity Outcomes

We used an HTT rat S1500+ dataset for 5-day exposure studies and extrapolated the normalized logarithmic counts per million reads to the whole transcriptome to increase the coverage of the altered gene signatures. Subsequently, we analyzed the extrapolated data using the traditional statistical method of analysis of variance (ANOVA) and looked at the results based on whether a chemical was classified as non-hepatotoxic (ACR (acrylamide); BDCA (bromodichloroacetic acid); THU (α,β-thujone); GIN (ginseng); EE2 (ethinyl estradiol); TBBPA (tetrabromobisphenol A); and MTE (milk thistle extract)) or hepatotoxic (COU (coumarin); DE71 (pentabromodiphenyl ether mixture); DEHP (di(2-ethylhexyl) phthalate); FEN (fenofibrate); FUR (furan); HCB (hexachlorobenzene); MET (methyl eugenol); PFOA (perfluorooctanoic acid); PUL (pulegone); TCAB (tris(chloropropyl) phosphate); and TCCP (3,3′,4,4′-tetrachloroazobenzene)). The one-way ANOVA results indicated that the majority of the non-hepatotoxic compounds did not show any genes that changed significantly (false discovery rate (FDR) < 0.1) compared to their controls (Supplementary Figure S1). Specifically, the chemicals ACR and BDCA showed the lowest numbers of significantly altered genes for any dose level, followed by THU and GIN. However, we did observe some abnormalities in dose–response behavior, with some of the dose levels for these chemicals showing large numbers of significantly altered genes in the middle dose range but not at the high dose levels. We believe this discrepancy could be due to variations in the guanine-cytosine content, sequencing depth, RNA-seq aligner, or normalization methods used by the HTT platform [14,15].

Compared to the chemicals (ACR, BDCA, THU, and GIN) that showed only a few significantly changing genes for any concentration, indicating their non-toxic behavior, we identified several significantly changing genes for the chemicals EE2, TBBPA, and MTE, although they were classified as non-hepatotoxic compounds. However, in contrast, we observed typical dose-dependent behavior for the number of significantly changed genes for all the hepatotoxic chemicals, i.e., a low number of significantly altered genes at lower concentrations that gradually increased as the dose level increased (Supplementary Figures S2 and S3), indicating major perturbations in liver metabolism, which suggests their potential for liver toxicity. Based on the total number of significantly altered genes, the results indicated that the S1500+ HTT platform captured dose–response behavior typical of any hepatotoxic chemical, where the number of significantly altered genes increased with dose, and it did not show any significant genes for some of the non-hepatotoxic chemicals. Although we consider this dose-dependent increase in the number of significantly altered genes for toxic chemicals as a surrogate to classify a chemical for potential toxicity, the major gene perturbations observed for the other three non-hepatotoxic chemicals indicate that the magnitude of gene perturbations alone may not be sufficient to ascertain liver toxicity and that additional parameters are required to differentiate them.

**Figure 1:**
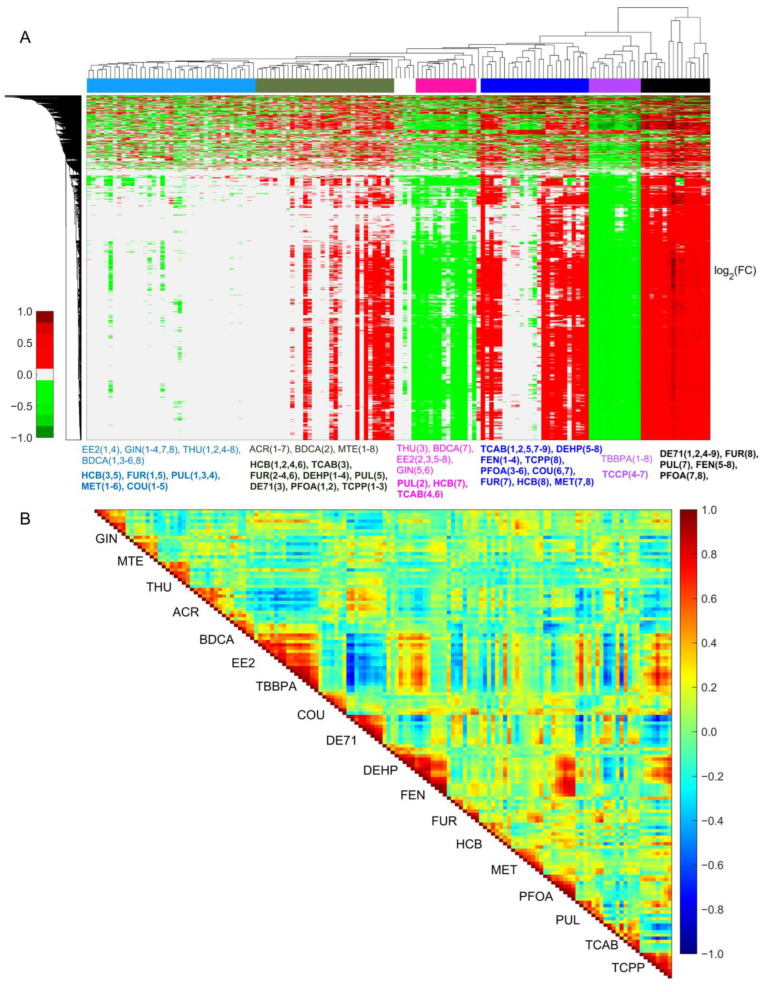
Cluster diagram and Pearson’s correlation coefficients of all genes across various doses and chemical exposures. (**A**) Hierarchical clustering of logarithmic fold-change (FC) values of all genes irrespective of their significance levels. Here, the columns represent the individual dose levels of each non-hepatotoxic and hepatotoxic chemical (name in bold font). Red and green indicate genes that are up- and downregulated, respectively. For clarity, concentrations of each chemical are shown as sequential numbers, with actual concentration values listed in Table 1. (**B**) Pairwise Pearson’s correlation coefficients of altered gene expression changes (independent of their significance levels) across all the dose ranges for the non-hepatotoxic and hepatotoxic chemicals.

To address whether the changes in gene fold-change values provide distinct expression patterns between non-hepatotoxic and hepatotoxic chemicals, we applied a hierarchical clustering analysis based on segregation by both genes and the dose levels of the chemicals. Figure 1A shows a clustergram plot of gene expression fold-change values without any significant cut-off values for all 18 chemicals with both row- and column-wise clustering. Clustering all chemicals without using any significance criteria allowed us to check gene expression changes across all chemicals to gain a quantitative understanding of the expression patterns between them and how they segregate with each other. For simplicity, we indicate the chemical dose levels using sequential numbers rather than the original concentration values in the plot (Table 1). We observed distinct gene expression patterns, with the majority of the non-hepatotoxic chemicals except ACR and BDCA segregated together for most of the dose levels, along with the low dose levels and some of the medium dose levels of the hepatotoxic chemicals (light blue and green bars in Figure 1A). Similarly, we observed that the hepatotoxic chemicals segregated together at the medium and highest dose levels, indicating potential common gene signatures that may suggest an impending liver injury. We observed a few exceptions with respect to the chemicals; for example, TBBPA and TCPP clustered together and showed irregular gene expression patterns, but the first chemical was classified as non-hepatotoxic, while the second was hepatotoxic. This behavior suggests that there could be alternative mechanisms underlying the gene expression patterns that indicate hepatotoxicity or that there is a specific small set of genes that are expressed similarly across all hepatotoxic compounds that characterize toxicity.

To quantify the degree of correlation between these chemicals, we calculated the pairwise Matthew’s correlation coefficient, as shown in Figure 1B. As expected, we observed a good correlation for the intra-dose levels of the same chemical and a reduced correlation between the chemicals. Specifically, we observed a significant correlation (>0.6) between the highest doses of the toxic chemicals that were segregated together in the clustergram analysis in Figure 1A. Furthermore, the gene fold-change values of the non-hepatotoxic chemicals EE2 and TBBPA were well correlated with some of the toxic chemicals, indicating that these chemicals may display potential toxic effects on liver metabolism at sufficiently high concentrations.

### 2.2. Significantly Changed Common Genes Differentiate Non-Hepatotoxic Chemicals from Hepatotoxic Chemicals

**Figure 2:**
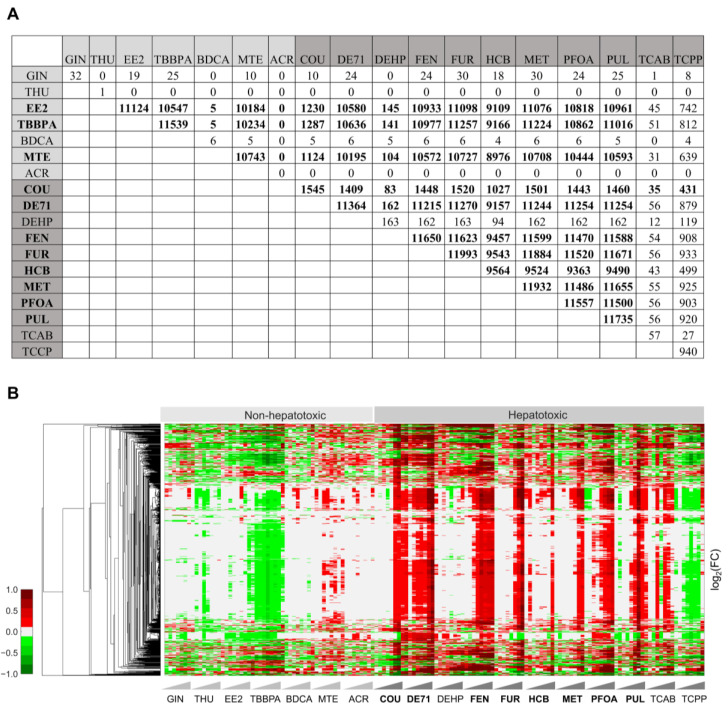
Genes with significant alterations and cluster diagram of genes that are common across highly toxic chemicals. (**A**) A summary of the total number of genes that changed significantly (false discovery rate < 0.1) at the highest concentration of each chemical and the common genes for their pairwise combinations. Here, the values in bold indicate the chemicals with the largest perturbations. (**B**) Hierarchical clustering of logarithmic fold-change (FC) values of significantly altered genes that are common for the highly toxic chemicals (indicated in bold font) and were monitored across all 18 chemicals. Red and green indicate genes that were up- and downregulated, respectively.

Although the hierarchical clustering analysis of the gene fold-change values for all the chemicals provided a potential toxic signature, many of these fold-change values were not statistically significant and, hence, may not be reliable predictors of hepatotoxicity. Therefore, we identified the genes that changed significantly at the highest dose for all the chemicals and conducted a pairwise comparison of the commonalities between them (Figure 2A). As indicated above, the majority of the non-hepatotoxic chemicals did not show any genes that changed significantly, except for EE2, TBBPA, and MTE. Similarly, among the hepatotoxic chemicals, we did not see a large number of genes that significantly changed for DEHP, TCAB, and TCCP when compared with other hepatotoxic chemicals (shown in bold in Figure 2A). The total number of changed genes was similar when we compared the highly changed hepatotoxic chemicals, and a pairwise mapping showed a large number of common genes, except for COU. Therefore, we identified significantly changed genes that were common to the highly toxic chemicals (Figure 2A, chemicals in bold font) and captured their gene expression pattern across all 18 chemicals.

Figure 2B shows a clustergram analysis of 941 common genes that are clustered by gene fold-change values across all 18 chemicals. We noted a distinct expression pattern for the non-hepatotoxic chemicals compared with the hepatotoxic chemicals, except for TCPP. The majority of the common genes were downregulated for the non-hepatotoxic chemicals, whereas they were upregulated for the hepatotoxic chemicals.

We observed clear dose–response behavior for the highly toxic chemicals (indicated in bold in Figure 2B), with downregulation at the low doses but upregulation as the concentrations increased. In contrast, for DEHP and TCCP, we observed a downregulation of the majority of these genes for most of the dose levels, except at the highest dose. However, the pairwise correlation coefficients for these chemicals showed a good correlation, and they correlated with the toxic chemicals FEN and PFOA (Supplementary Figure S4). These results suggest that the hepatotoxicity of TCCP may not be apparent until its highest dose and that it might cause toxicity through a slightly different mechanism compared to the other hepatotoxic chemicals in this study. We performed an additional KEGG enrichment analysis using these common genes at the highest concentration (Supplementary Table S1) to ascertain in which pathways these common genes belong and how they are altered between non-toxic and hepatotoxic chemicals. Our results indicated that several pathways involved in carbohydrate, lipid, amino acid, cofactor, and vitamin metabolism were significantly upregulated for the hepatotoxic chemicals (Supplementary Figure S5). Interestingly, at the highest dose level, the pathways altered for TCPP were similar to the other hepatotoxic chemicals, indicating its potential mechanism of toxicity. Taken together, our analysis of the significantly changed genes across the highly toxic chemicals provided a common set of genes and pathways that differentiates hepatotoxic chemicals with increased correlation (~0.8) for some of the high doses, and these genes and pathways can be used as potential gene signatures to assess hepatotoxicity with further validation.

### 2.3. Alterations of Pathways in Organismal, Environmental, and Metabolism Processes Are Potential Indicators of Toxicity

To understand the effect of dose at the pathway level, we performed a KEGG pathway enrichment analysis to understand the biological significance of changes in gene expression across all dose levels. We used the aggregated fold-change (AFC) method implemented in the web tool ToxPanel [16] to calculate z-score values that indicate whether a particular KEGG-annotated pathway was up- or downregulated. We provide a complete summary of the enriched pathways for all non-hepatotoxic and hepatotoxic chemicals in Supplementary Tables S2 and S3, respectively. For the KEGG-defined organismal processes, such as the endocrine system and immune system, for non-hepatotoxic chemicals, we observed that the majority of these pathways were downregulated for GIN, EE2, and TBBPA, whereas they were upregulated for MTE and THU (Supplementary Table S2). A similar comparison for the hepatotoxic chemicals indicated that many of these immune- and endocrine-related pathways were downregulated across all the chemicals except TCAB (Supplementary Table S3). In particular, the peroxisome proliferator-activated receptor (PPAR) signaling pathway was significantly upregulated across all the hepatotoxic chemicals compared to the non-hepatotoxic chemicals. Because PPAR signaling pathways are involved in the regulation of glucose and energy homeostasis and play a major role in lipid-accumulation-related processes, the disruption of this pathway for the hepatotoxic chemicals indicated that it may be one of the mechanisms that leads to liver toxicity [17]. Similarly, when we monitored KEGG environmental processes, such as signal transduction, signaling molecules, and their interaction, we observed that the majority of signal transduction pathways were downregulated for GIN and TBBPA, but they were upregulated for MTE (Supplementary Table S2) and did not change significantly for the other non-hepatotoxic chemicals. In contrast, the majority of these signal transduction pathways were downregulated for hepatotoxic chemicals at high doses, indicating that they are potential toxic mechanisms that lead to liver injury for these chemicals (Supplementary Table S3). The downregulation for chemicals classified as non-hepatotoxic, such as GIN and TBBPA, indicated potential hepatotoxicity for these chemicals, and the upregulation observed for MTE indicated its potential hepatoprotective role.

**Figure 3:**
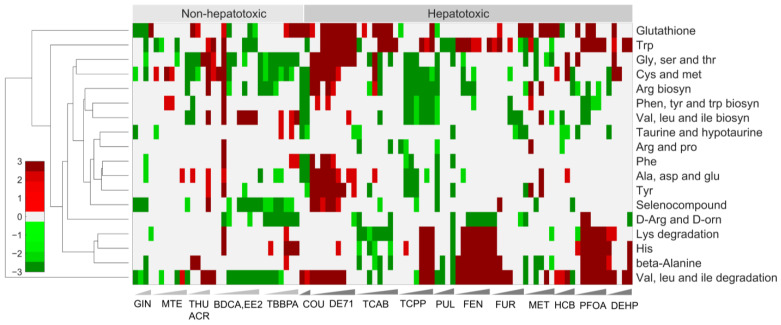
Cluster diagrams of alterations in the KEGG pathways related to amino acid metabolism. Hierarchical clustering of individual subpathways in amino acid metabolism for both non-hepatotoxic chemicals and hepatotoxic chemicals. Red and green indicate pathways that are up- and downregulated, respectively.

The liver plays a major role in various metabolism-related processes, such as lipid, amino acid, carbohydrate, vitamin, and xenobiotic metabolism [18]. The majority of the non-hepatotoxic chemicals showed either no changes for most of the dose levels in the amino acid-related processes or upregulation for some of the high doses for ACR and BDCA (Figure 3). In contrast, we noted downregulation for the non-hepatotoxic chemicals GIN, EE2, and TBBPA. In particular, these chemicals downregulated valine, leucine, and isoleucine degradation [19]; cysteine and methionine [20]; as well as glycine, serine, and threonine metabolic pathways across several dose levels. Cysteine and glycine are precursors to glutathione synthesis, which plays a major role in chemical-induced oxidative stress, and the downregulation of these pathways, together with the downregulation of the glutathione pathway, indicated the non-hepatotoxic behavior observed for these chemicals. In contrast, we observed a significant upregulation of the valine, leucine, and isoleucine degradation pathways across multiple doses of hepatotoxic chemicals (Figure 3 and Supplementary Table S3). Similarly, as reported in a previous study [21], we observed an upregulation of glycine- and cysteine-related pathways across several doses, although it was not consistent across all the hepatotoxic chemicals and dose levels. However, we observed a significant upregulation of the glutathione pathway across multiple dose levels of the hepatotoxic chemicals, indicating oxidative-stress-related mechanisms that lead to liver toxicity [22]. Our results for other pathways in amino acid metabolism, such as the lysine degradation and beta-alanine-, tryptophan-, and histidine-metabolism-related processes that were all upregulated for hepatotoxic chemicals compared to non-hepatotoxic chemicals, indicated potential molecular pathways to monitor for liver toxicity, in addition to the glutathione as well as valine, leucine, and isoleucine pathways [10].

**Figure 4:**
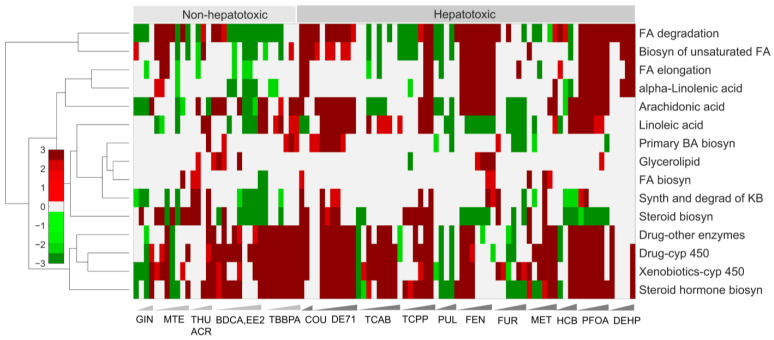
Cluster diagrams of alterations in the KEGG pathways related to lipid metabolism. Hierarchical clustering of individual subpathways in lipid metabolism for both non-hepatotoxic and hepatotoxic chemicals. Red and green indicate pathways that are up- and downregulated, respectively.

The liver also plays a key role in lipid metabolism and is involved in fatty acid synthesis; lipid circulation through lipoprotein synthesis; and lipid storage, including triglyceride synthesis and the formation of lipid droplets [23]. Disruptions in lipid metabolism and degradation-related pathways, such as beta oxidation and fatty acid synthesis, lead to lipid accumulation and are the hallmark of non-alcoholic fatty liver disease [24]. Our pathway enrichment analysis indicated that some of the major lipid-related pathways, such as the synthesis and degradation of ketone bodies, fatty acid degradation, and arachidonic acid metabolism pathways, were significantly downregulated for GIN, EE2, and TBBPA, whereas they were upregulated for the other non-hepatotoxic chemicals (Figure 4). We found a similar upregulation behavior for the hepatotoxic chemicals for these three lipid-related pathways, indicating that these pathways may not be large contributors to the hepatotoxic behavior (Figure 4). However, when we examined the biosynthesis of unsaturated fatty acids, fatty acid elongation, and alpha-linolenic acid metabolism, these pathways were either unchanged or downregulated for the non-hepatotoxic chemicals but were significantly upregulated for the hepatotoxic chemicals, indicating they play a major role in lipid-related disruptions that cause liver toxicity. However, these changes were not entirely consistent across all of the hepatotoxic chemicals, indicating the potential for alternative mechanisms (Figure 4 and Supplementary Tables S2 and S3).

### 2.4. Toxicant-Induced Changes in Gene Expression Predict Liver Histopathological Phenotypes

Using our previously developed liver injury modules, we tested the predictive capability of the altered gene expression changes for estimating potential liver histopathological outcomes [25]. We used the logarithmic fold-change values of all the genes for each chemical compared to their corresponding control groups (no significant cut-off values were used) and calculated the liver injury module z-score values based on the absolute AFC method (see Materials and Methods) [16]. We then calculated the activation of the injury modules for each dose and considered the modules with the largest z-score values with aggregated *p*-values less than 0.05 as the most probable histopathological phenotypes for each chemical. We provide a summary of all the calculated z-score values for each chemical and each dose in Supplementary Table S4.

**Figure 5:**
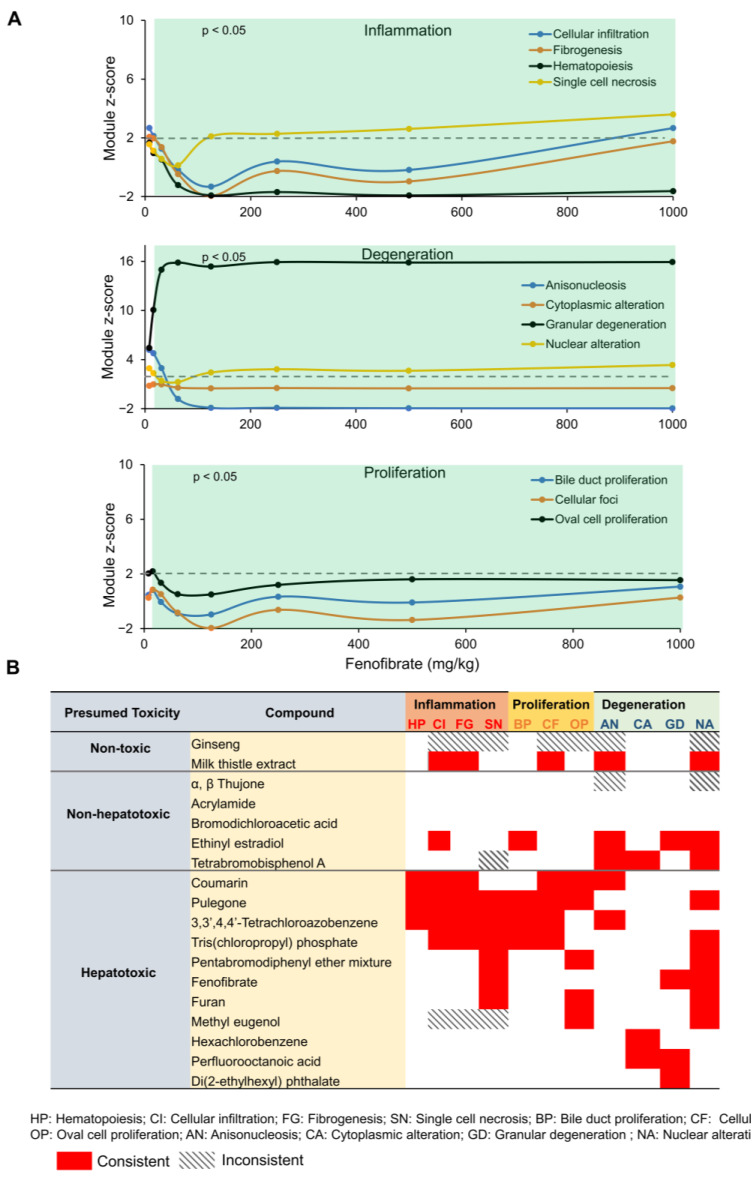
Summary of the liver histopathological outcomes for all 18 chemicals. (**A**) An exemplar summary of the dose–response behavior of injury module activation scores for the chemical fenofibrate (FEN). Green shaded region indicates the dose levels for which the z-score values are significant (*p* < 0.05). (**B**) An overall summary of the liver injury module predictions of the histopathological outcomes for all 18 chemicals classified as non-toxic, non-hepatotoxic, and hepatotoxic. We indicate a module is activated with the color red, and this is consistent for each chemical if the z-score values are greater than 2 and are significant with *p*-values less than 0.05 for at least two consecutive dose levels (Supplementary Table S4). If the z-score values are greater than 2 and significant for low dose levels but not significant at high dose levels (*p*-value > 0.05), then the module activation is considered inconsistent (striped bar).

Figure 5A shows an exemplar summary of the dose–response behavior of injury module activation scores for the chemical FEN. To facilitate easy interpretation of the individual histopathological endpoints, we grouped the injury phenotypes into three overarching cellular processes: inflammation, proliferation, and degeneration. We considered an injury module with an activation score greater than 2 (indicated by the dashed line in Figure 5A) and a *p*-value less than 0.05 as a significantly altered histopathological outcome for a given dose level. For example, in Figure 5A, the top plot shows the activation scores for histopathological endpoints in the inflammation phenotype, and FEN consistently activated single-cell necrosis as the potential injury endpoint. However, we found an exponential increase in the granular degeneration endpoint (middle plot in Figure 5A) with an increasing dose level, indicating that FEN predominantly activated the injury process of degeneration as a major histopathological outcome. We expected this result because FEN was part of the large set of chemicals used to develop the liver injury modules in our original study, where granular degeneration was the experimental histopathological outcome [25,26,27]. This result shows that the 5-day rat study using the S1500+ platform was able to capture the histopathological outcomes effectively from the extrapolated gene expression changes.

Figure 5B shows a summary of the liver histopathological outcomes for all 18 chemicals. Here, we retained the original classification by which the chemicals were divided into non-toxic, non-hepatotoxic, and hepatotoxic based on the chronic studies [13]. As described above (Figure 5A), for a module to be activated, we selected the criteria of a z-score greater than 2 and a *p*-value less than 0.05 and indicated the histopathological outcome in red if it was activated consistently for at least two dose levels. If the module was activated only by magnitude (z-score > 2) but was not statistically significant, then we indicated that with a striped bar. We did not observe any significant activation of the liver injury modules for GIN, THU, ACR, or BDCA, hence their status as non-hepatotoxic chemicals. However, we did observe the activation of liver injury modules for MTE, EE2, and TBBPA. Historically, MTE was used as a liver-protective agent [28,29], and currently our liver injury modules are not able to differentiate protective and toxic gene expression signatures. Similarly, our liver injury modules predicted activation for the two non-hepatotoxic chemicals EE2 and TBBPA. Previous studies indicated the activation of several gene-ontology-based liver biological processes for these two chemicals, and liver toxicity was indicated in some of the human hormonal therapy and mouse studies, indicating their potential to cause liver toxicity at high concentrations [13,30,31].

In the case of the hepatotoxic chemicals, the liver injury modules significantly activated multiple cellular processes for each chemical (Figure 5B). The majority of the hepatotoxic chemicals significantly activated several histopathological endpoints in cellular inflammation and proliferation processes. For example, COU [32], PUL [33], and TCAB [34] commonly activated hematopoiesis, cellular infiltration, fibrogenesis, and cellular foci, whereas TCCP, DE71, FEN, and FUR activated single-cell necrosis and nuclear alteration as the major histopathological endpoints [35]. In contrast, MET and HCB [36] activated only nuclear alteration in the proliferation process as the major histopathological endpoint, while PFOA and DEHP activated only degeneration-related processes. These results suggest that despite differences in their molecular structures and mechanisms of chemical toxicity, our injury modules showed that these chemicals activate several common injury processes that ultimately lead to liver toxicity. Our predictions are supported by several studies in the literature that predicted similar histological outcomes for the majority of the hepatotoxic chemicals [33,34,35,37,38,39,40,41].

## 3. Materials and Methods

### 3.1. Animals and Chemical Selection

All the animal experiments were approved by the Battelle Animal Care and Use Committee and were conducted in accordance with all relevant National Institutes of Health and NTP animal care and use policies. A detailed description of the complete methods is available as part of the original study [13]. Briefly, 8- to 10-week-old male Sprague Dawley rats were exposed to eight or nine concentrations of each of the 18 selected chemicals, along with a control group. The chemicals were administered via oral gavage (5 mL/kg) once per day for 5 consecutive days (Days 0–4), with four animals for each dose and a vehicle control. The dose levels for each chemical were selected based on the concentrations used in the chronic and sub-chronic toxicity study and included concentrations that were higher and lower than those previously used [13]. Table 1 shows all the chemicals, their dose levels, and their corresponding vehicle controls used in this study. On Day 5, the rats were killed via exsanguination and the liver and kidney tissues were collected. The left liver lobes and right kidneys were transferred into RNAlater (Qiagen, Valencia, CA, USA) in accordance with the manufacturer’s guidelines for RNA extraction.

### 3.2. Liver RNA Extraction and HTT Using the Rat S1500+

Total RNA was extracted from the liver with a DNA digestion step using an RNeasy Mini Kit (Qiagen). After RNA purity and quality checks using the absorbance ratio (260/280) and the RNA integrity number, respectively, the samples were frozen and sent to BioSpyder (Carlsbad, CA, USA) for HTT analysis using the rat S1500+ TempO-Seq platform [12]. The mRNA targets were hybridized with a detector oligo (DO) mix for amplification and barcoded for proper identification following sequencing. The prepared libraries were then sequenced using the HiSeq 2500 Ultra-High-Throughput Sequencing System (Illumina, San Diego, CA, USA), and readouts were demultiplexed to generate FASTQ files.

After an initial quality control analysis of the FASTQ files, the sequences were then aligned to the probe sequences from the target platform using Bowtie version 1.2.2 [42]. This configuration allowed up to three mismatches and reported the single best alignment. After alignment, the total sequenced reads, the percentage of reads aligning with the platform manifest, the alignment rate, and the percentage of expressed probes (≥5 reads per probe) were calculated for each sample. Samples were flagged for values below the following thresholds: sequencing depth < 500 K, total alignment rate < 40%, unique alignment rate < 30%, number of aligned reads < 500 K, and percentage of probes with at least five reads < 50%. Filtering based on the percentage of expressed probes eliminated biased samples for which the sequenced reads only reflected a small portion of the measured transcriptome. Furthermore, principal component, hierarchical cluster, and inter-replicate correlation analyses were performed to determine which samples were outliers so they could be removed from further downstream analyses. Subsequently, the aligned read counts for attenuated probes were properly readjusted to calculate unattenuated equivalent counts using the attenuation factors provided in the platform manifest. To account for between-sample sequencing depth variation, unattenuated read counts were normalized at the probe level by applying reads-per-million normalization. A pseudo-read-count of 1.0 was added to each normalized expression value. Then, the values were log2 transformed to complete the normalization.

Finally, the normalized log-transformed values from the S1500+ dataset were then used for extrapolation to the whole transcriptome (~19 K genes) using a commercial platform (GeniE, version 3.0.4) [12]. This approach incorporated PC regression [43] and was updated to use roughly 20 K samples of publicly available rat transcriptomics data from the Gene Expression Omnibus (GEO) and Short Read Archive (SRA) to train the rat model and a large collection of publicly available RNA-seq data [44] to train the human model. The filtering criteria for the training data required samples to meet the following minimum thresholds: 1,000,000~aligned reads, non-zero expression for 25% of whole transcriptome genes, and non-zero expression for 25% of species-specific S1500+ genes. Samples were also required to have 90% of the total reads mapped to at least 1000 genes. The training dataset included 18,699 genes.

### 3.3. Gene Expression, KEGG Pathway, and Liver Injury Module Analysis

To identify differentially expressed genes from the extrapolated log-transformed whole-transcriptomic data, we performed a one-way ANOVA and estimated the FDR to correct for multiple comparisons. We defined a significantly expressed gene as one with an FDR-adjusted *p*-value of ≤0.05. We also used our previously developed AFC and aggregate absolute fold-change methods (AAFC) to identify gene sets that significantly changed between the treatment and control conditions [16,45,46]. For the KEGG pathway enrichment analysis, we used the AFC method, and it provided directionality of gene expression in terms of whether the pathway was up- or downregulated. Briefly, the AFC method calculated the mean fold-change (FC) value for each gene and defined the KEGG pathway score as the total FC value of all genes in the pathway. The sign of the pathway score represented the direction of regulation, with positive values indicating upregulation and negative values indicating downregulation in the treatment condition compared to their corresponding controls.

We used the AAFC method to calculate the activation scores of liver injury (histopathology) modules that were significantly changed [16,46]. It provided a measure of how much a set of genes was disrupted, regardless of whether the genes were over- or under-expressed. Briefly, the AAFC method first calculated the absolute value of each gene’s log-transformed FC value and then calculated the total FC value of the absolute values for each module. We then used the gene set scores to perform null hypothesis tests and estimated each gene set’s significance using its *p*-value, defined as the probability that the score for randomly selected FC values (10,000 times) was greater than the score from the actual gene set. A small *p*-value implied that the gene set value was significant. The z-score was the number of standard deviations by which the actual gene set value differed from the mean of the randomly selected FC values (10,000 times). In this study, we used all the genes, irrespective of their significance criteria for each chemical, to determine the module activation scores.

## 4. Conclusions

There is an imminent need to assess a large number of environmental chemicals for their potential toxicity and to provide adequate countermeasures to protect human health. As a result, there has been a substantial effort to develop rapid, cost-efficient, high-throughput methods that can screen thousands of chemicals and characterize their potential toxicity. These newly developed HTT platforms provide a screening approach for estimating safe chemical exposure doses that produce minimal biological activity [11,12]. In this work, we used the HTT platform and a 5-day in vivo rat model using 18 environmental and industrial chemicals with the objective of evaluating whether the HTT-derived altered gene expression changes from the chemical exposures can predict hepatotoxicity outcomes to differentiate between hepatotoxic and non-toxic chemicals and predict potential histopathological injury-initiating processes that indicate liver injury.

Our results show that the extrapolated whole-transcriptomic chemical exposure data obtained using the HTT platform provided potential altered gene signatures with distinct expression patterns that differentiated hepatotoxic from non-hepatotoxic chemicals. Specifically, we identified a set of 941 common genes that were significantly altered (FDR < 0.1), with a high correlation across the highly hepatotoxic chemicals, and showed that the majority of the genes were upregulated for the hepatotoxic chemicals compared to the non-hepatotoxic chemicals. In addition, indicative of the potential mechanism of toxicity, we identified several molecular pathways in the endocrine and immune systems, such as the complement and coagulation cascades (downregulated) and the PPAR signaling pathways (upregulated) [47,48], respectively, that were uniquely altered for hepatotoxic chemicals compared to non-toxic chemicals. Similarly, in the signal transduction pathways, we identified pathways, such as FoxO signaling and Jak-STAT signaling [49], that were significantly downregulated only for hepatotoxic chemicals (Supplementary Table S3). Furthermore, our analysis also indicated several metabolism-related processes in amino acid metabolism, such as valine, leucine, and isoleucine metabolism; cysteine and methionine metabolism; tryptophan metabolism; and glutathione metabolism, that were upregulated only for hepatotoxic chemicals (Figure 3 and Supplementary Figure S5). Similarly, we identified pathways associated with lipid metabolism, such as the biosynthesis of unsaturated fatty acids, fatty acid elongation, and alpha-linolenic acid metabolism, that were significantly upregulated only for the hepatotoxic chemicals (Figure 4), indicating potential molecular initiating processes that lead to liver toxicity. Importantly, our study provided high-level description and identification of liver histopathological phenotypes based on changes in gene expression using the liver injury module analysis. Our results showed clear inflammatory and proliferation responses for the majority of the chemicals, which matched with prior experimental studies. Taken together, these results indicated that the changes in gene expression obtained using the HTT platform provided a reasonable estimate of hepatotoxicity at multiple levels of cellular complexity and provided potential histopathological outcomes. Therefore, the proposed platform can be used as an approach to characterize new chemicals to understand their mechanisms of toxicity and potential liver injury phenotypes that might lead to end-stage liver disease.

## Figures and Tables

**Table 1:** Summary of the chemicals used in the 5-day rat study and their dose levels and vehicle controls.

Chemical	Gavage Vehicle	Concentrations *								
		1	2	3	4	5	6	7	8	9
ACR	Deionized water	0.078	0.156	0.3125	0.625	1.25	2.5	5	10	
BDCA	Deionized water	1.25	2.5	5	10	20	40	80	160	
COU	Corn oil	3.125	6.25	12.5	25	50	100	20	400	
DEHP	Corn oil	8	16	31.25	62.5	125	250	500	1000	
DE71	Corn oil	0.38	0.75	1.5	3	15	50	100	200	500
EE2	Corn oil	0.02	0.067	0.2	0.6	1.8	5.4	16.2	48.6	
FEN	0.5% aqueous methylcellulose	8	16	31.25	62.5	125	250	500	1000	
FUR	Corn oil	0.125	0.25	0.5	1	2	4	8	16	
GIN	Deionized water	39.1	78.125	156.25	312.5	625	1250	2500	5000	
HCB	Corn oil	0.004	0.015	0.0625	0.25	1	4	16	64	
MET	0.5% aqueous methylcellulose	4.625	9.25	18.5	37	75	150	300	600	
MTE	Corn oil	39.1	78.125	156.25	312.5	625	937.5	1250	1750	
PFOA	2% Tween 80	0.156	0.3125	0.625	1.25	2.5	5	10	20	
PUL	Corn oil	2.4	4.7	9.4	18.75	37.5	75	150	300	
TBBPA	Corn oil	4	8	16	125	250	500	1000	2000	
TCAB	Corn oil/acetone (99:1)	0.1	0.3	1	3	10	30	100	200	400
TCPP	0.5% aqueous methylcellulose	18.75	37.5	75	150	300	600	1000	2000	
THU	0.5% aqueous methylcellulose	1.5	3	6.25	12.5	25	50	100	200	

ACR: acrylamide; BDCA: bromodichloroacetic acid; COU: coumarin; DEHP: di(2-ethylhexyl) phthalate; DE71: pentabromodiphenyl ether mixture; EE2: ethinyl estradiol; FEN: fenofibrate; FUR: furan; GIN: ginseng; HCB: hexachlorobenzene; MET: methyl eugenol; MTE: milk thistle extract; PFOA: perfluorooctanoic acid; PUL: pulegone; TBBPA: tetrabromobisphenol A; TCAB: tetrachloroazobenzene; TCCP: tris(chloropropyl) phosphate; THU: α,β-thujone. * Units: mg/kg for all the chemicals except EE2 (µg/kg).

## Data Availability

The datasets presented in this study are derived from the original study which is openly available in the NCBI’s GEO database gene repository for rats under accession number GSM4415261. The derived datasets supporting the conclusions of this article will be made available by the authors on request.

## Supplementary Materials

The following supporting information can be downloaded at https://www.mdpi.com/1422-0067/24/24/17425/s1.

## References

[1] Waters M.D., Fostel J.M. (2004). Toxicogenomics and systems toxicology: Aims and prospects. Nat. Rev. Genet..

[2] Ramaiahgari S.C., Auerbach S.S., Saddler T.O., Rice J.R., Dunlap P.E., Sipes N.S., DeVito M.J., Shah R.R., Bushel P.R., Merrick B.A. (2019). The Power of Resolution: Contextualized Understanding of Biological Responses to Liver Injury Chemicals Using High-throughput Transcriptomics and Benchmark Concentration Modeling. Toxicol. Sci..

[3] Spellman P.T., Sherlock G., Zhang M.Q., Iyer V.R., Anders K., Eisen M.B., Brown P.O., Botstein D., Futcher B. (1998). Comprehensive identification of cell cycle-regulated genes of the yeast *Saccharomyces cerevisiae* by microarray hybridization. Mol. Biol. Cell.

[4] Aardema M.J., MacGregor J.T. (2002). Toxicology and genetic toxicology in the new era of “toxicogenomics”: Impact of “-omics” technologies. Mutat. Res..

[5] Kullak-Ublick G.A., Andrade R.J., Merz M., End P., Benesic A., Gerbes A.L., Aithal G.P. (2017). Drug-induced liver injury: Recent advances in diagnosis and risk assessment. Gut.

[6] Stine J.G., Chalasani N.P. (2017). Drug Hepatotoxicity: Environmental Factors. Clin. Liver Dis..

[7] Kiyosawa N., Ando Y., Manabe S., Yamoto T. (2009). Toxicogenomic biomarkers for liver toxicity. J. Toxicol. Pathol..

[8] Lauschke V.M. (2021). Toxicogenomics of drug induced liver injury—From mechanistic understanding to early prediction. Drug Metab. Rev..

[9] Pannala V.R., Estes S.K., Rahim M., Trenary I., O’Brien T.P., Shiota C., Printz R.L., Reifman J., Shiota M., Young J.D. (2020). Toxicant-Induced Metabolic Alterations in Lipid and Amino Acid Pathways Are Predictive of Acute Liver Toxicity in Rats. Int. J. Mol. Sci..

[10] Schyman P., Printz R.L., Pannala V.R., AbdulHameed M.D.M., Estes S.K., Shiota C., Boyd K.L., Shiota M., Wallqvist A. (2021). Genomics and metabolomics of early-stage thioacetamide-induced liver injury: An interspecies study between guinea pig and rat. Toxicol. Appl. Pharmacol..

[11] Judson R.S., Mortensen H.M., Shah I., Knudsen T.B., Elloumi F. (2012). Using pathway modules as targets for assay development in xenobiotic screening. Mol. Biosyst..

[12] Mav D., Shah R.R., Howard B.E., Auerbach S.S., Bushel P.R., Collins J.B., Gerhold D.L., Judson R.S., Karmaus A.L., Maull E.A. (2018). A hybrid gene selection approach to create the S1500+ targeted gene sets for use in high-throughput transcriptomics. PLoS ONE.

[13] Gwinn W.M., Auerbach S.S., Parham F., Stout M.D., Waidyanatha S., Mutlu E., Collins B., Paules R.S., Merrick B.A., Ferguson S. (2020). Evaluation of 5-day In Vivo Rat Liver and Kidney with High-throughput Transcriptomics for Estimating Benchmark Doses of Apical Outcomes. Toxicol. Sci..

[14] Bushel P.R., Paules R.S., Auerbach S.S. (2018). A Comparison of the TempO-Seq S1500+ Platform to RNA-Seq and Microarray Using Rat Liver Mode of Action Samples. Front. Genet..

[15] Everett L.J., Mav D., Phadke D.P., Balik-Meisner M.R., Shah R.R. (2022). Impact of Aligner, Normalization Method, and Sequencing Depth on TempO-seq Accuracy. Bioinform. Biol. Insights.

[16] Schyman P., Xu Z., Desai V., Wallqvist A. (2021). TOXPANEL: A Gene-Set Analysis Tool to Assess Liver and Kidney Injuries. Front. Pharmacol..

[17] Tyagi S., Gupta P., Saini A.S., Kaushal C., Sharma S. (2011). The peroxisome proliferator-activated receptor: A family of nuclear receptors role in various diseases. J. Adv. Pharm. Technol. Res..

[18] Rui L. (2014). Energy metabolism in the liver. Compr. Physiol..

[19] Lo E.K.K., Felicianna, Xu J.H., Zhan Q., Zeng Z., El-Nezami H. (2022). The Emerging Role of Branched-Chain Amino Acids in Liver Diseases. Biomedicines.

[20] Pastore A., Alisi A., di Giovamberardino G., Crudele A., Ceccarelli S., Panera N., Dionisi-Vici C., Nobili V. (2014). Plasma levels of homocysteine and cysteine increased in pediatric NAFLD and strongly correlated with severity of liver damage. Int. J. Mol. Sci..

[21] Pannala V.R., Vinnakota K.C., Rawls K.D., Estes S.K., O’Brien T.P., Printz R.L., Papin J.A., Reifman J., Shiota M., Young J.D. (2019). Mechanistic identification of biofluid metabolite changes as markers of acetaminophen-induced liver toxicity in rats. Toxicol. Appl. Pharmacol..

[22] Vairetti M., Di Pasqua L.G., Cagna M., Richelmi P., Ferrigno A., Berardo C. (2021). Changes in Glutathione Content in Liver Diseases: An Update. Antioxidants.

[23] Nguyen P., Leray V., Diez M., Serisier S., Le Bloc’h J., Siliart B., Dumon H. (2008). Liver lipid metabolism. J. Anim. Physiol. Anim. Nutr..

[24] Pei K., Gui T., Kan D., Feng H., Jin Y., Yang Y., Zhang Q., Du Z., Gai Z., Wu J. (2020). An overview of lipid metabolism and nonalcoholic fatty liver disease. BioMed Res. Int..

[25] Tawa G.J., AbdulHameed M.D., Yu X., Kumar K., Ippolito D.L., Lewis J.A., Stallings J.D., Wallqvist A. (2014). Characterization of chemically induced liver injuries using gene co-expression modules. PLoS ONE.

[26] Uehara T., Ono A., Maruyama T., Kato I., Yamada H., Ohno Y., Urushidani T. (2010). The Japanese toxicogenomics project: Application of toxicogenomics. Mol. Nutr. Food Res..

[27] Zubrzycki A., Wronska A., Kotulak-Chrzaszcz A., Wierzbicki P.M., Kmiec Z. (2020). Fenofibrate impairs liver function and structure more pronounced in old than young rats. Arch. Gerontol. Geriatr..

[28] Tamayo C., Diamond S. (2007). Review of clinical trials evaluating safety and efficacy of milk thistle (*Silybum marianum* [L.] Gaertn.). Integr. Cancer Ther..

[29] Hackett E.S., Twedt D.C., Gustafson D.L. (2013). Milk thistle and its derivative compounds: A review of opportunities for treatment of liver disease. J. Vet. Intern. Med..

[30] Pandey G., Pandey S.P., Sharma M. (2011). Experimental hepatotoxicity produced by ethinyl estradiol. Toxicol. Int..

[31] Yao L., Wang Y., Shi J., Liu Y., Guo H., Yang X., Liu Y., Ma J., Li D., Wang Z. (2021). Toxicity of tetrabromobisphenol A and its derivative in the mouse liver following oral exposure at environmentally relevant levels. Environ. Sci. Technol..

[32] Tanaka Y., Fujii W., Hori H., Kitagawa Y., Ozaki K. (2016). Relationship between coumarin-induced hepatocellular toxicity and mitochondrial function in rats. Food Chem. Toxicol..

[33] Ribeiro-Silva C.M., Faustino-Rocha A.I., Gil da Costa R.M., Medeiros R., Pires M.J., Gaivao I., Gama A., Neuparth M.J., Barbosa J.V., Peixoto F. (2022). Pulegone and eugenol oral supplementation in laboratory animals: Results from acute and chronic studies. Biomedicines.

[34] van Birgelen A.P., Hebert C.D., Wenk M.L., Grimes L.K., Chapin R.E., Mahler J., Travlos G.S., Bucher J.R. (1999). Toxicity of 3,3’,4,4’-tetrachloroazobenzene in rats and mice. Toxicol. Appl. Pharmacol..

[35] Dunnick J.K., Pandiri A.R., Merrick B.A., Kissling G.E., Cunny H., Mutlu E., Waidyanatha S., Sills R., Hong H.L., Ton T.V. (2018). Carcinogenic activity of pentabrominated diphenyl ether mixture (DE-71) in rats and mice. Toxicol. Rep..

[36] Giribaldi L., Chiappini F., Pontillo C., Randi A.S., Kleiman de Pisarev D.L., Alvarez L. (2011). Hexachlorobenzene induces deregulation of cellular growth in rat liver. Toxicology.

[37] Thorup I., Wurtzen G., Carstensen J., Olsen P. (1983). Short term toxicity study in rats dosed with pulegone and menthol. Toxicol. Lett..

[38] (1998). NTP Technical Report on the Toxicity Studies of 3,3’,4,4’-Tetrachloroazobenzene (CAS No. 14047-09-7) Administered by Gavage to F344/N rats and B6C3F1 mice. Toxic Rep. Ser..

[39] Moser G.J., Foley J., Burnett M., Goldsworthy T.L., Maronpot R. (2009). Furan-induced dose-response relationships for liver cytotoxicity, cell proliferation, and tumorigenicity (furan-induced liver tumorigenicity). Exp. Toxicol. Pathol..

[40] Dong H., Gill S., Curran I.H., Williams A., Kuo B., Wade M.G., Yauk C.L. (2016). Toxicogenomic assessment of liver responses following subchronic exposure to furan in Fischer F344 rats. Arch. Toxicol..

[41] National Toxicology Program (2000). NTP Toxicology and Carcinogenesis Studies of Methyleugenol (CAS NO. 93-15-2) in F344/N rats and B6C3F1 mice (Gavage Studies). Natl. Toxicol. Program Tech. Rep. Ser..

[42] Langmead B., Trapnell C., Pop M., Salzberg S.L. (2009). Ultrafast and memory-efficient alignment of short DNA sequences to the human genome. Genome Biol..

[43] Jolliffe I.T. (2018). A Note on the Use of Principal Components in Regression. J. R. Stat. Soc. Ser. C Appl. Statist..

[44] Lachmann A., Torre D., Keenan A.B., Jagodnik K.M., Lee H.J., Wang L., Silverstein M.C., Ma’ayan A. (2018). Massive mining of publicly available RNA-seq data from human and mouse. Nat. Commun..

[45] Yu C., Woo H.J., Yu X., Oyama T., Wallqvist A., Reifman J. (2017). A strategy for evaluating pathway analysis methods. BMC Bioinform..

[46] Schyman P., Printz R.L., Estes S.K., Boyd K.L., Shiota M., Wallqvist A. (2018). Identification of the toxicity pathways associated with thioacetamide-induced injuries in rat liver and kidney. Front. Pharmacol..

[47] Liss K.H., Finck B.N. (2017). PPARs and nonalcoholic fatty liver disease. Biochimie.

[48] Qiu Y.Y., Zhang J., Zeng F.Y., Zhu Y.Z. (2023). Roles of the peroxisome proliferator-activated receptors (PPARs) in the pathogenesis of nonalcoholic fatty liver disease (NAFLD). Pharmacol. Res..

[49] Negi C.K., Khan S., Dirven H., Bajard L., Blaha L. (2021). Flame Retardants-Mediated Interferon Signaling in the Pathogenesis of Nonalcoholic Fatty Liver Disease. Int. J. Mol. Sci..

