# Correction: Pannala et al. High-Throughput Transcriptomics Differentiates Toxic versus Non-Toxic Chemical Exposures Using a Rat Liver Model. *Int. J. Mol. Sci.* **2023**, *24*, 17425

**DOI:** 10.3390/ijms25137108

**Published:** 2024-06-28

**Authors:** Venkat R. Pannala, Michele R. Balik-Meisner, Deepak Mav, Dhiral P. Phadke, Elizabeth H. Scholl, Ruchir R. Shah, Scott S. Auerbach, Anders Wallqvist

**Affiliations:** 1 Department of Defense Biotechnology High Performance Computing Software Applications Institute, Telemedicine and Advanced Technology Research Center, U.S. Army Medical Research and Development Command, Fort Detrick, Frederick, MD 21702, USA; 2 The Henry M. Jackson Foundation for the Advancement of Military Medicine, Inc., Bethesda, MD 20817, USA; 3 Sciome LLC, Research Triangle Park, Durham, NC 27709, USA; michele.balik-meisner@sciome.com (M.R.B.-M.); deepak.mav@sciome.com (D.M.); dhiral.phadke@sciome.com (D.P.P.); elizabeth.scholl@sciome.com (E.H.S.); ruchir.shah@sciome.com (R.R.S.); 4 Division of Translational Toxicology, National Institute of Environmental Health Sciences, Research Triangle Park, Durham, NC 27709, USA; auerbachs@niehs.nih.gov

In the original publication [1], the following authors were not included. The newly included authors (Michele R. Balik-Meisner, Deepak Mav, Dhiral P. Phadke, Elizabeth H. Scholl, Ruchir R. Shah, and Scott S. Auerbach) were involved in data curation, processing, and quality control along with writing—review and editing of the published article. The affiliations, author contributions statement, data availability statement, and conflicts of interest have been corrected accordingly.

There were also errors in the second and third paragraphs of Section 3.2, including an incorrect description of some of the parameters used in the RNA-sequencing data analysis and in the S1500+ data extrapolation to the whole transcriptome using GeniE software. In Section 3.2, on Page 12, the second paragraph, Lines 7 to 9, the sentence should be changed to the following: sequencing depth < 500 K, total alignment rate < 40%, unique alignment rate < 30%, number of aligned reads < 500 K, and percentage of probes with at least five reads < 50%. In Section 3.2, on Page 12, the third paragraph, Lines 1 to 5, the sentence should be changed to the following: Finally, the normalized log-transformed values from the S1500+ dataset were then used for extrapolation to the whole transcriptome (~19 K genes) using a commercial platform (GeniE, version 3.0.4) [12]. This approach incorporated PC regression [43] and was updated to use roughly 20 K samples of publicly available rat transcriptomics data from the Gene Expression Omnibus (GEO) and Short Read Archive (SRA) to train the rat model and a large collection of publicly available RNA-seq data [44] to train the human model. In Section 3.2, on Page 12, the third paragraph, Line 7, the percentage (35%) should be changed to 25%. In Section 3.2, on Page 12, the third paragraph, Line 9, the number of genes (25,599) should be changed to 18,699.

The corrected author contributions statement, data availability statement, conflicts of interest, appear here.

**Author Contributions:** Conceptualization, V.R.P. and A.W.; methodology and analysis, V.R.P.; data curation, processing, and quality control, M.R.B.-M., D.M., D.P.P., E.H.S., R.R.S. and S.S.A.; writing—original draft preparation, V.R.P.; writing—review and editing, V.R.P., M.R.B.-M., D.M., D.P.P., E.H.S., R.R.S., S.S.A. and A.W.; supervision, A.W.; funding acquisition, A.W. All authors have read and agreed to the published version of the manuscript.
**Data Availability Statement:** The datasets presented in this study are derived from the original study which is openly available in the NCBI’s GEO database gene repository for rats under accession number GSM4415261. The derived datasets supporting the conclusions of this article will be made available by the authors on request.
**Conflicts of Interest:** V.R.P. is employed by The Henry M. Jackson Foundation for the Advancement of Military Medicine, Inc., and M.R.B.-M., D.M., D.P.P., E.H.S., and R.R.S. are employed by Sciome LLC. The remaining authors declare that the research was conducted in the absence of any commercial or financial relationships that could be construed as potential conflicts of interest.

The authors state that the scientific conclusions are unaffected. This correction was approved by the Academic Editor. The original publication has also been updated.
