# Supplementary material for: High-Throughput Transcriptomics Differentiates Toxic versus Non-Toxic Chemical Exposures Using a Rat Liver Model
Source: Int J Mol Sci. 2023 Dec 13;24(24):17425. doi: 10.3390/ijms242417425 (PMC10743995; doi:10.3390/ijms242417425)
Supplement: Supplementary file 1 [file ijms-24-17425-s001.zip › 12_6_2023MK Pannala_IJMS_2023_supplementary_material.pdf]

Supplementary Material

High-throughput transcriptomics differentiates toxic versus non-toxic chemical exposures on rat liver metabolism

Venkat R. Pannala[1,2,*] and Anders Wallqvist[1,*]

[1]Department of Defense Biotechnology High Performance Computing Software Applications Institute, Telemedicine and Advanced Technology Research Center, U.S. Army Medical Research and Development Command, Fort Detrick, MD, USA
[2]The Henry M. Jackson Foundation for the Advancement of Military Medicine, Inc., Bethesda, MD, USA

*Correspondence:
Venkat Pannala
Email: vpannala@bhsai.org
Tel.: (301) 619-1978
Anders Wallqvist
Email: sven.a.wallqvist.civ@health.mil
Tel.: (301) 619-1989

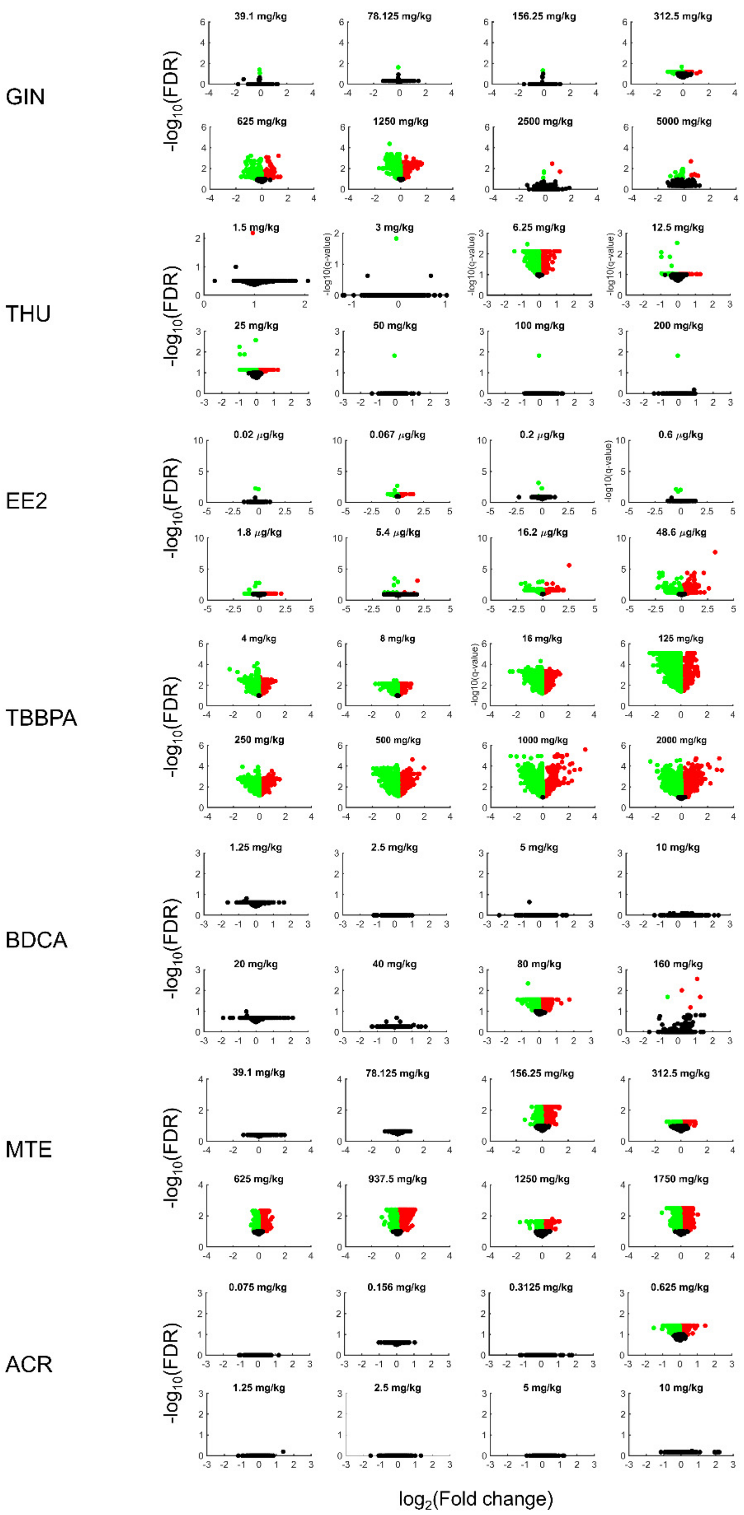

Figure S1: Volcano plots for the changes in gene expression for the non-hepatotoxic chemicals. Red and green circles indicate genes that were significantly [false discovery rate (FDR) < 0.1] up- and downregulated, respectively.

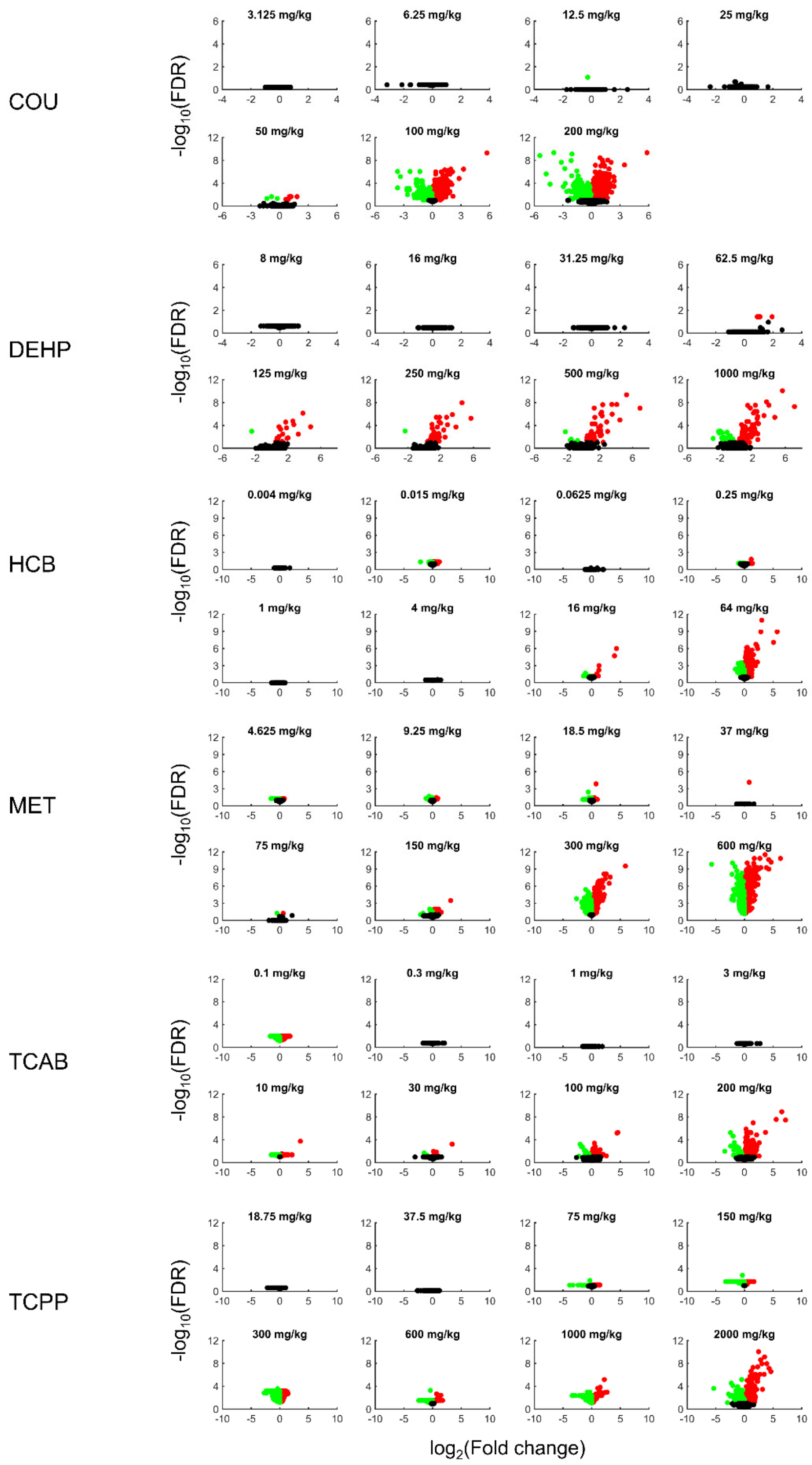

Figure S2: Volcano plots for the changes in gene expression for the hepatotoxic chemicals shown on left side of the figure. Red and green circles indicate genes that were significantly [false discovery rate (FDR) < 0.1] up- and downregulated, respectively.

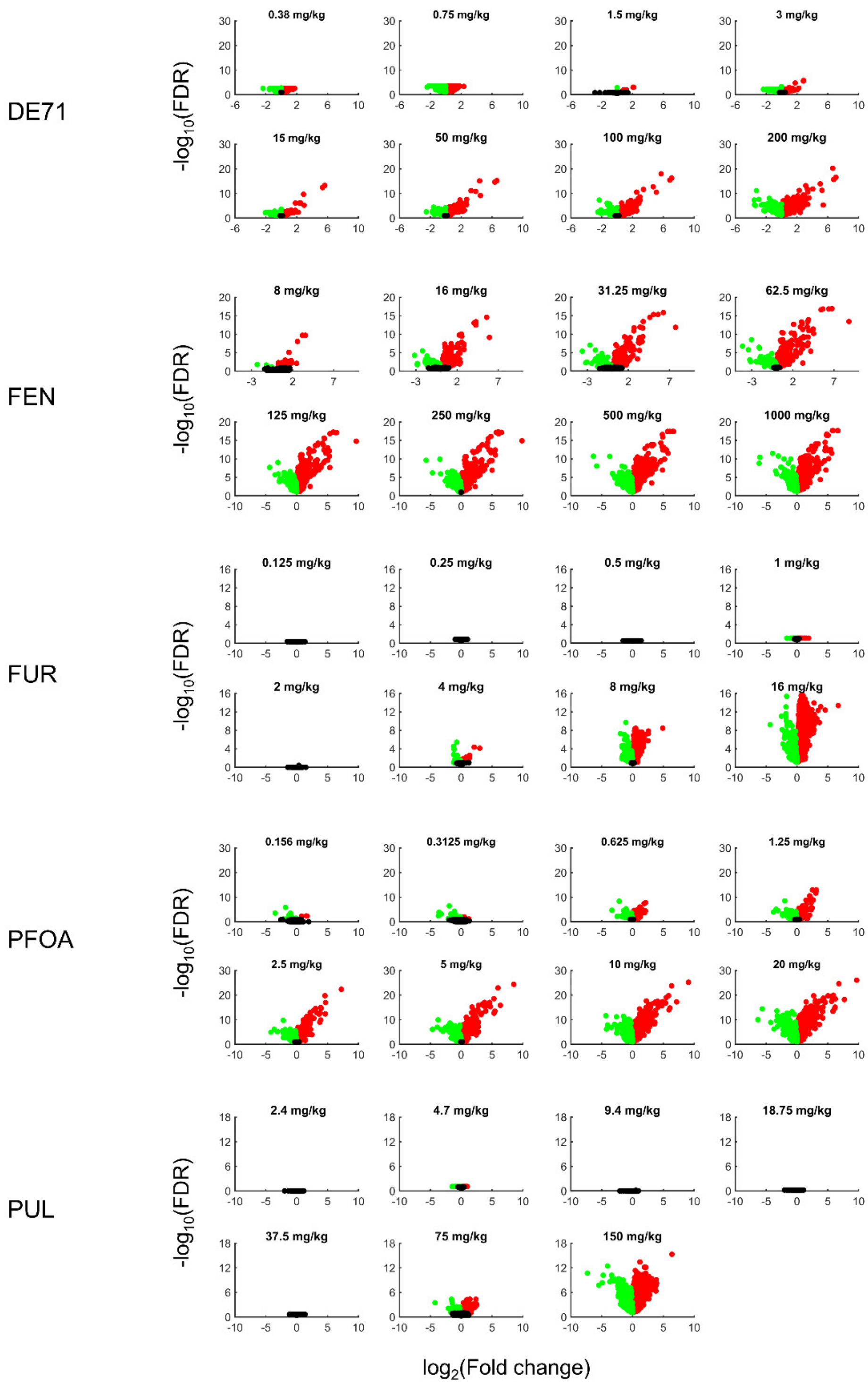

Figure S3: Volcano plots for the changes in gene expression for the hepatotoxic chemicals shown on left side of the figure. Red and green circles indicate genes that were significantly [false discovery rate (FDR) < 0.1] up- and downregulated, respectively.

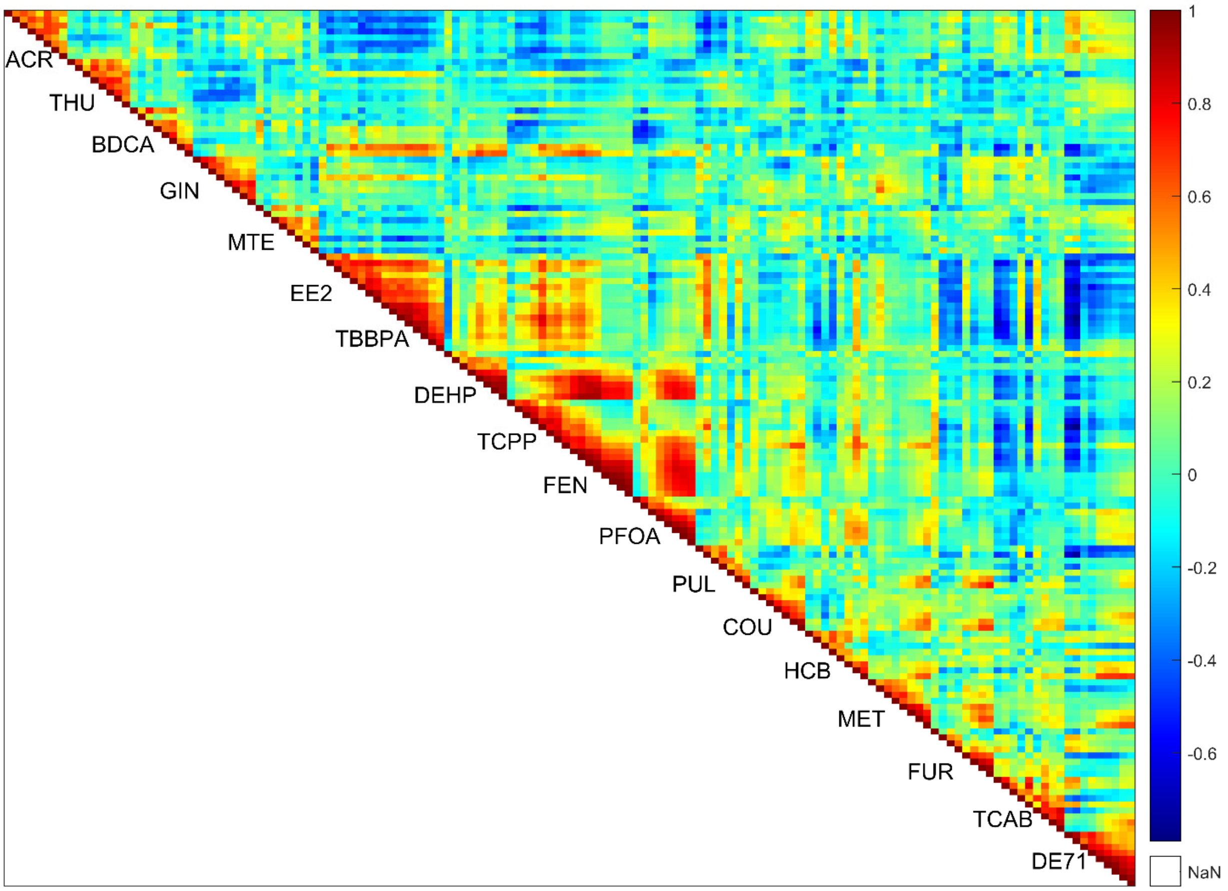

Figure S4: Pairwise Pearson's correlation coefficients based on significantly altered genes for all chemicals. The significant genes were selected based on genes common to hepatotoxic chemicals at the highest dose.

KEGG Pathway	GIN	EE	MTE	TBBPA	COU	DE71	DEHP	FEN	FUR	HCB	MET	PFOA	PUL	TCAB	TCCP
						AFC z-score values									
Metabolic pathways	-2.2	0.2	2.1	0.8	1.9	4.2	6.7	5.1	-2.7	4.2	4.5	5.7	-3.6	2.1	7.3
Carbon metabolism	-0.4	-1.3	3.9	-2.7	1.9	1.6	5.8	5.2	0.7	0.0	1.5	4.0	0.4	1.3	4.0
Ascorbate and aldarate metabolism	0.9	6.5	-0.1	4.8	3.8	3.0	5.3	1.3	0.0	3.5	2.9	2.5	-2.2	8.8	7.7
Pentose and glucuronate interconversions	0.6	6.5	0.6	4.7	4.2	3.6	5.3	1.4	0.6	3.8	4.1	2.7	-1.7	8.9	8.4
Butanoate metabolism	0.3	-0.9	-0.1	0.6	-0.3	-1.9	5.9	4.3	-2.4	-2.6	-1.0	2.3	-3.1	-2.2	3.1
Pyruvate metabolism	0.1	-3.1	1.8	-0.3	2.1	1.4	2.1	1.7	0.4	0.6	1.0	0.7	0.3	2.2	2.5
Propanoate metabolism	0.9	-2.1	0.3	0.7	1.2	-0.4	5.6	4.0	0.3	-0.6	0.5	2.5	-0.3	-0.5	3.9
Nitrogen metabolism	-1.2	-2.3	-1.5	0.0	-3.6	-3.2	-1.9	-2.0	-2.0	-2.4	-1.8	-5.3	-5.9	-3.9	-2.2
Arachidonic acid metabolism	-4.0	-1.0	-4.5	2.7	-1.6	4.3	0.9	0.7	-3.0	7.3	4.6	3.0	-0.7	-2.9	3.2
Fatty acid elongation	0.0	-0.2	0.1	-0.4	1.5	-1.7	5.0	4.8	0.1	-0.1	-0.1	3.8	-1.5	-2.0	3.1
Fatty acid metabolism	-0.6	-3.5	0.6	0.6	2.2	-0.5	10.5	10.9	-1.1	-1.4	0.5	8.5	-2.2	-1.8	6.6
Fatty acid degradation	-0.8	-3.7	0.8	0.7	2.2	-0.4	10.0	10.6	-1.0	-1.3	0.6	8.3	-2.2	-1.7	6.1
Linoleic acid metabolism	-2.4	-0.6	-2.3	0.7	-7.0	4.2	-2.3	-6.5	-7.2	4.8	-0.4	-1.2	-8.5	1.4	-0.3
Steroid hormone biosynthesis	-1.5	3.0	-1.8	7.3	-0.8	6.9	4.5	-0.8	-5.9	8.3	4.2	3.7	-6.9	5.7	7.8
Biosynthesis of unsaturated fatty acids	-1.5	-1.9	0.5	-0.1	2.6	0.1	8.7	8.5	-0.2	0.4	0.9	7.0	-1.6	-1.5	5.6
Tryptophan metabolism	1.0	-2.0	0.2	-0.5	-2.8	0.9	2.4	1.7	-2.6	-0.3	-0.7	0.0	-3.7	2.5	0.8
Histidine metabolism	1.0	1.3	-0.3	1.3	0.6	1.1	6.1	5.2	-0.2	1.3	-0.4	5.4	-0.2	-0.7	2.3
beta-Alanine metabolism	0.3	-0.1	-0.9	0.7	0.5	-1.7	7.4	5.2	-0.6	-1.5	-0.3	4.7	-1.7	-2.6	4.0
Valine, leucine and isoleucine degradation	-0.9	-3.4	1.3	0.2	1.8	0.5	8.9	7.6	-1.3	-1.1	0.6	6.3	-1.8	-1.4	5.6
alpha-Linolenic acid metabolism	-1.8	-2.6	0.6	1.2	2.7	1.7	8.9	8.6	-0.5	0.5	1.3	7.8	-1.4	-0.4	6.3
Lysine degradation	0.7	-2.8	-0.2	0.3	0.7	-1.5	6.7	4.6	-1.1	-2.1	-0.3	2.4	-1.3	-1.5	4.2
Glutathione metabolism	-0.3	-1.5	-0.7	1.6	4.0	4.9	-0.3	-1.2	2.3	2.5	4.6	-0.7	2.3	4.8	3.5
Retinol metabolism	-1.5	3.8	-1.7	8.2	0.6	8.6	5.7	0.1	-4.4	10.8	6.5	5.4	-5.5	6.8	10.5
Porphyrin and chlorophyll metabolism	1.1	7.0	0.0	5.9	4.2	3.7	5.9	1.7	0.0	3.8	3.1	3.2	-2.2	9.8	8.2
Drug metabolism - other enzymes	2.3	4.1	-0.5	6.5	5.9	6.0	4.8	0.3	0.9	4.7	5.2	2.0	-0.9	10.4	8.3
Drug metabolism - cytochrome P450	2.2	4.0	0.1	6.6	5.3	7.7	4.1	-0.7	0.5	6.2	5.8	1.2	-1.7	13.9	8.2
Metabolism of xenobiotics by cytochrome P450	-0.1	2.9	1.3	5.9	6.8	9.7	4.0	0.7	2.6	7.8	8.6	2.0	-0.3	14.0	9.4
IL-17 signaling pathway	-1.0	2.5	1.8	0.0	-2.0	4.0	-2.6	0.2	-0.9	-2.2	-0.3	-2.9	3.1	0.4	-2.6
ABC transporters	0.2	0.9	-0.4	2.3	3.9	3.8	0.1	0.9	5.1	1.8	6.7	0.7	5.5	0.6	2.8
Caffeine metabolism	0.2	-0.8	-0.4	-1.1	-2.4	2.9	-2.6	-3.4	-2.4	2.2	-0.6	-2.8	-3.3	6.3	-2.3
Peroxisome	-1.1	-2.5	0.1	1.6	2.1	0.2	12.0	10.5	-1.0	-0.8	1.3	9.2	-2.9	-1.6	6.9
Progesterone-mediated oocyte maturation	5.1	-0.7	-3.9	3.1	1.8	-0.3	0.9	-1.5	4.5	2.8	-0.6	-2.0	1.5	2.2	1.5
Complement and coagulation cascades	-2.1	-2.6	1.3	-0.7	-2.2	0.3	-2.1	-1.6	-3.2	-0.9	-1.3	-1.8	-2.5	-1.0	-1.8
Bile secretion	-1.4	-1.0	1.3	1.2	3.3	4.2	-0.4	0.1	4.0	2.5	6.8	-0.3	4.5	1.3	3.7
PPAR signaling pathway	-2.3	-2.2	2.1	0.2	2.4	0.7	7.9	8.9	0.1	0.0	1.5	6.8	-1.5	-0.3	4.9
Serotonergic synapse	-2.2	-0.8	-2.0	-0.5	-4.5	0.5	-1.1	-3.5	-4.6	1.4	-0.2	-0.4	-4.7	-2.7	-1.4
Chemical carcinogenesis	-0.7	2.5	-2.0	9.0	3.5	11.5	4.9	0.2	-2.0	11.7	8.9	4.6	-3.4	9.7	10.9
Pathways in cancer	-0.1	-1.1	2.2	-0.5	3.1	2.5	-0.7	-2.0	2.2	1.6	3.9	-1.2	1.3	2.3	1.4
Cell cycle	4.2	-0.3	-3.1	2.5	3.0	-0.9	0.4	-1.3	5.3	2.7	0.1	-1.6	2.8	1.4	0.8
Fluid shear stress and atherosclerosis	0.7	-1.4	1.0	1.6	3.7	5.8	-0.8	-1.3	1.7	2.7	4.6	-0.6	1.3	5.4	2.7

Figure S5: KEGG pathway enrichment analysis for significantly altered genes common to hepatotoxic chemicals at the highest dose. Values in red and green indicate aggregate fold-change (AFC) z-scores that were significantly [false discovery rate (FDR) < 0.1] up- and downregulated, respectively.
